# DNA Methyltransferases in Depression: An Update

**DOI:** 10.3389/fpsyt.2020.538683

**Published:** 2020-09-03

**Authors:** Zhenghao Duan, Jie Lu

**Affiliations:** ^1^Department of Human Anatomy, College of Basic Medical Sciences, China Medical University, Shenyang, China; ^2^Department of Neurology, Shengjing Hospital of China Medical University, Shenyang, China

**Keywords:** depression, DNA methylation, DNA methyltransferase, neurodevelopment, DNMT3L, DNMT3A, DNMT3B, DNMT1

## Abstract

Depression is one of the most common psychiatric disorders affecting public health. Studies over the past years suggest that the methylations of some specific genes such as *BDNF*, *SLC6A4*, and *NR3C1* play an important role in the development of depression. Recently, epigenetic evidences suggest that the expression levels of DNA methyltransferases differ in several brain areas including the prefrontal cortex, hippocampus, amygdala, and nucleus accumbens in depression patients and animal models, but the potential link between the expression levels of DNA methylatransferases and the methylations of specific genes needs further investigation to clarify the pathogenesis of depression.

## Highlights

1. We did a thorough analysis of progress of this field on the basis of literature search in Pubmed for the last 5 years.
2. We proposed a new model of gene-environment interaction through DNA methylation, connecting the missing links between stressors and depression signs, which could be used as potential targets for the depression therapy.
3. We suggested some important questions for the further exploration in this field, especially on *DNMT3L*, a little studied modulator of *de novo* DNA methylation during brain development, its expression relatively rich in hippocampus and amygdala, which are important brain areas for depression.

## Introduction

Depression, also known as major depressive disorder (MDD), is a common chronic and recurrent mental disorder characterized by at least two weeks of presenting with low mood and aversion to daily activities, which affect normal social life (1). In 2017, the prevalence counts of MDD were about 163 million people, together with other depressive disorders, making them the third largest group of disease burden in the world (2). There are many hypotheses regarding the pathogenesis of depression, including those with neurotransmitters (e.g., biogenic amines), genetic, endocrine, and inflammatory mechanistic bases, but no single one can explain all aspects of the depression (3).

Recent studies support a gene-environment interaction model, where epigenetic modifications are the key connectors, whose changes may cause the gene expression alteration in different pathways involved in this disorder, as a way of adaptation (4). These epigenetic modifications include DNA methylation and demethylation, histone acetylation and deacetylation, non-coding RNA, and chromatin remodeling, among which DNA methylation is the most stable modification that could be passed to the next generation, and DNA methyltransferases (DNMTs) are critical enzymes whose activities underlie these processes. Growing evidences suggest that DNA methylation and DNMTs are involved in the development of depression (5). DNMTs expression changes have been found in different brain areas in depression patients (6, 7) and animal models (8, 9). Among these studies, frontal cortex and amygdala are the common affected areas in both human and animal, suggesting DNMTs may contribute to the cognitive and emotional domains of depression endophenotypes, which are also cortisol-related (10, 11). The other affected areas include hypothalamus in depression patients and hippocampus and nucleus accumbens in animal models. However, we still do not know, how are these DNMTs regulated in the neural circuits of depression, and what are the target genes of these DNMTs in this scenario.

In the current review, we summarized the recent progress on DNMTs studies in depression, discussed the controversies in this field, and provided potential directions for further exploring the mechanism of DNMTs in depression. We hope it can help us to understand the pathogenesis of depression, as well as provide a new perspective for the targeted therapy of depression.

## DNA Methylation

DNA methylation, one of the most important epigenetic modifications, was first discovered in 1940s (12). DNA methylation mainly occurs at cytosine nucleotides in DNA sequence where a cytosine nucleotide is followed by a guanine nucleotide (CG sites), though non-CG sites methylation also exists (13, 14). The methylation in promoter region is usually associated with the repression of gene expression, whereas the methylation in the gene body region may be associated with increased gene expression (12). The process involves the addition of a methyl group from the cofactor SAM (S-adenosyl-methionine) to the 5th carbon of the cytosine residue to form 5mC and it is catalyzed by DNMTs. The 5mC can be actively demethylated by ten–eleven translocations (TETs) or activation-induced cytidine deaminase/apolipoprotein B mRNA-editing enzyme complex (AID/APOBEC) enzymes, together with thymine DNA glycosylase (TDG) (12). This methylation modification can physically impede the binding of transcription factors to the gene itself, or by recruiting protein complexes, contribute to many physiological processes such as genomic imprinting, X-chromosome inactivation, and the regulation of the chromatin structure and gene expression (12). However, an increasing number of studies suggest the mechanisms of how DNA methylation regulates gene expression are more complicated than we have expected.

## DNA Methyltransferases

The catalysis and maintenance of DNA methylation depend on DNMTs. In mammals, DNMTs include DNMT1, DNMT2, and DNMT3. DNMT3 includes DNMT3A, DNMT3B, and DNMT3L (15). DNMT2 only catalyzes RNA methylation, which uses tRNA as its substrate, and has no DNA methylation activity (16). So the DNMTs that participate in DNA methylation consist of DNMT1, DNMT3A, DNMT3B, and DNMT3L. DNMT1 maintains DNA methylation while DNMT3A and DNMT3B help *de novo* DNA methylation (12). DNMT3L can’t catalyze DNA methylation directly, but it can modulate the DNA methylation through activating DNMT3A and DNMT3B (17, 18).

The function of DNMTs in the brain has been investigated in *Dnmt1/Dnmt3a* knockout mice, where *Dnmt1* knockout in embryonic brain led to demethylation of the *Gfap* promoter in neural precursor cells, promoting astrogliosis (19, 20), and *Dnmt1/Dnmt3a* double knockouts in forebrain postmitotic neurons caused the deficits in synaptic plasticity and learning and memory (21). The expressions of DNMTs in the developing brain are different among mice (21), rats (22), primates (23), and human (24), but for most DNMTs, they share a common pattern that the expression peaks in early development and declines in adult, high in proliferating cells and low in differentiated cells.

## DNA Methylation in Depression

Many of the studies on DNA methylation in depression used animal models. There are several approaches to induce the depression-like behaviors in rodents, such as early life stressors, corticosterone supplementation, learned helplessness, social defeat stress, and genetic modification (25). These approaches can usually generate behavioral phenotypes analogous to depression signs required for depression diagnosis, especially anhedonia (25, 26). Although it is difficult to mimic the depression signs in animals due to its heterogeneous nature, there are some consensual depression-like behavioral tests being used by researchers in this field to model the signs of depression, such as the coat state assessment (CSA) and sucrose consumption test (SCT) for anhedonia-like behavior, the restraint stress test (RST), the tail suspension test (TST) and the forced swim test (FST) for helplessness and despair-like behavior, the novel object recognition (NOR), spatial memory test (SMT) and the Morris water maze (MWM) for cognitive changes, the open field test (OFT) for locomotor and anxiety-like behavior, and the affective bias test (ABT) for reward learning and processing (25, 27–29). However, given the heterogeneity of this disease and the limitations of animal models, results from these studies should be interpreted with caution (26).

Recent studies demonstrated that the methylations of some specific genes such as *P11* (30–33), *BDNF* (34–38), *SLC6A4* (39–42), and *NR3C1* (43–45), were closely correlated to depression. For example, Svenningsson et al. found that the *P11* knockout mice showed anhedonia-like and despair-like behaviors assessed with above mentioned SCT, TST, and FST (33). When the *P11* gene was re-introduced into the nucleus accumbens of the *P11* knockout mice, the anhedonia-like and despair-like behaviors could be effectively eliminated (33). Seo et al. found that P11 knockdown in the lateral habenula alleviated the stress-induced anhedonia-like and despair-like behaviors in rats, while overexpressing P11 in dopamine D2 receptor-containing lateral habenula neurons of control mice induced anhedonia-like and despair-like behaviors assessed with SCT, RST, TST, and FST (32).

With the progress of methylation measurement, there are increasing numbers of clinical studies showing the correlation between the gene methylation changes and depression symptoms. For example, one study found the hypermethylation of *BDNF* promoter in the peripheral blood cells was correlated with the severity of the depression (34). Another clinical study found similar changes of *BDNF* hypermethylation in the buccal tissue of depression patients (36). Serotonin dysregulation is one of the most studied pathways in depression. In a monozygotic twin study, Zhao et al. found the DNA methylation levels of the *SLC6A4* promoter were positively correlated with the Beck Depressive Inventory scores for depressive symptoms (42). In another study, Lam et al. found *SLC6A4* DNA methylation was associated with depression status in the presence of specific genotype (40). Glucocorticoid receptor (GR) is another well-studied molecule in depression based on stress model. Melas et al. found that the hypermethylation changes of the promoter of GR gene *NR3C1* were correlated with childhood adversities in depression patients (43). Na et al. found decreased *NR3C1* methylation changes in non-psychotic depression patients (45). Additionally, several methylome-wide association studies have shown global DNA methylation changes in depression patients, with enriched genes involved in neurodevelopment (5).

In summary, DNA methylation might be the essential link between the environment and gene expression in depression, but its detailed mechanism needs thorough research. Though some results are contradictory to each other, most studies support the hypothesis that the hypermethylation changes in specific genes induce decreased expressions in depression patients or animal models and all these changes are involved in the pathways related to depression (5, 46). However, we do not know exactly what causes the methylation changes and how the genes are specifically targeted for the methylation changes. We can speculate that environmental changes (stressors) during brain development may activate the hypothalamic–pituitary–adrenal (HPA) axis, releasing glucocorticoids, which can activate the immediate early genes (IEGs) through GRs; the IEGs then stimulate the expressions of DNMTs. The DNMTs target specific genes assisted by transcription factors (TFs) and non-coding RNAs (ncRNAs), whose activation may represent a stress response or plasticity in cells, imitating a stem cell state when DNA methylation is active. These specific gene expression changes can affect the balance of neurotransmitters and therefore cause depression symptoms. This is supported by the observation that the DNA methylation was dynamically regulated, and environmental enrichment in adolescent rats that were exposed to adversity stressors rescued the DNA methylation profiles and recovered their typically observed behavior in non-treated animals (47), and DNA methylation inhibitor zebularine normalized the aberrant behavior in FST observed in rats exposed to maltreatment during infancy (48).

## DNA Methyltransferases in Depression

### DNMT1

DNMT1 was first discovered in 1988, which is involved in the maintenance of DNA methylation (49, 50). It was found that the mRNA level of DNMT1 was decreased in the peripheral blood of patients with depression in attacking stage, but there was no difference between depression patients in remission stage and healthy subjects (51). The results in postmortem patients with depression also showed that the expression levels of DNMT1 mRNA in the prefrontal lobe and amygdala were decreased, which was the same as that in the peripheral blood (6).

However, contrary to above findings, the experiments in animal models showed opposite results. Morris et al. found that mice with *Dnmt1* knockout in forebrain showed anxiolytic and antidepressant-like effects assessed with elevated plus maze (EPM), FST, and novelty suppressed feeding test (NSF) (52). Melas et al. discovered that treating depressive rats with escitalopram could down-regulate the expression of Dnmt1 in prefrontal cortex (31). And in hippocampus, genipin reduced despair-like traits (assessed *via* TST and FST) by inhibiting the expression of Dnmt1 and normalizing the expression of BDNF (53). Additionally, Wright et al. found that the expression of Dnmt1 was increased in the central nucleus of amygdala in the depression mouse model established by chronic social failure stress (assessed *via* a social interaction test (SIT)), but only in the female mice (54). Park et al. used male rats to establish the depression model through maternal deprivation. It was found that the expression levels of Dnmt1 were significantly increased in the hippocampus of depression-like rats, and decreased after antidepressant treatment with escitalopram (55).

In the depression animal model established by maternal deprivation and social isolation, Dnmt1 expression and *Nr3c1* promoter methylation level of female rats were higher than those in male rats. It is believed that early life stress destroyed the protective effect of estrogen. This is consistent with the current high prevalence of depression among women, but it is not clear whether it can be seen as the principle cause of the difference in the incidence of depression between men and women (55). It is still not known what causes this discrepancy; the DNMT1 expression might be tissue specific and age and sex dependent, which need deliberate investigation.

### DNMT3A and DNMT3B

DNMT3A and DNMT3B have direct catalytic activity for DNA methylation, as functional DNA methyltransferases. DNMT3B plays an important role in the process of early brain development and neurogenesis whereas DNMT3A is more essential to mature neuron than DNMT3B (21, 56). According to a postmortem study from suicide patients with MDD, there was no significant change of DNMT3A expression in the prefrontal cortex, amygdala, and paraventricular nucleus of hypothalamus, but the DNMT3B expressions were increased in the prefrontal cortex and paraventricular nucleus and decreased in amygdala of patients compared to healthy controls (6). In another study, the DNMT3B expressions in the dorsolateral prefrontal cortex of depression patients were increased, and the DNMT3A expressions showed no change compared to those in healthy controls (7). This is consistent with a study done with peripheral white blood cells (51).

Although no correlation was found between DNMT3A and depression in clinical studies, it is different in the animal models. Several studies have shown a causal relationship between Dnmt3a and depression, but the results are not consistent. In one of the studies, Elliott et al. found that the depressive mice established by chronic social defeat expressed lower Dnmt3a in the medial prefrontal cortex than wild type mice did (57). The *Dnmt3a*-knockout mice also had anxiety-like behavior [assessed *via* OFT, dark-light transfer (DLT), EPM, and home cage locomotion], whereas Dnmt3a overexpression in the medial prefrontal cortex induced an anxiolytic effect. However, in another study, Sales et al. found adult rats underwent learned helplessness displayed despair-like behavior (assessed *via* numbers of escape failures) accompanied by an increment of Dnmt3a and Dnmt3b expressions in medial prefrontal cortex and that treatment with antidepressant imipramine down-regulated their expression levels in the prefrontal cortex (9). Melas et al. also demonstrated that Dnmt3a expression level was decreased in prefrontal cortex after the depressive rats (Flinders Sensitive Line) (58) were treated with antidepressant escitalopram (31). This discrepancy may reflect the different depression models and behavioral tests used under the same depression umbrella, but it also indicates the significance of DNMT3A in all the models.

Since different brain areas may play different roles in the pathogenesis of depression, it is not surprising that Dnmt3a alterations are different in these areas. Besides prefrontal cortex, Dnmt3a changes were also found in nucleus accumbens and hippocampus in animal experiments. One study showed that Dnmt3a expressions in the nucleus accumbens were increased after the rats underwent social chronic defeats and Dnmt3a overexpressions in the nucleus accumbens promoted the occurrence of depression-like behaviors (social avoidance and despair-like behavior assessed *via* SIT and FST) (59). In another study, exposure to subchronic variable stress induced depression-associated behaviors (assessed *via* splash test, SCT, FST, EPM, and locomotor activity) and increased the expressions of Dnmt3a in the nucleus accumbens of mice, but the expression levels were higher in female mice than those in male mice (60). The different sensitivities of mice to depression were determined by the expression of Dnmt3a in the nucleus accumbens. After Dnmt3a coding genes in male mice were enhanced in the nucleus accumbens, the sensitivities to subchronic variable stress were increased, which were similar to those observed in female mice. Oppositely, after Dnmt3a genes were specifically knocked out in female mice, their adaptabilities to subchronic variable stress were significantly enhanced, which were similar to those of male mice (60).

Critical for learning and memory and mood regulation, hippocampus is the brain region associated with cognitive and emotional domains of depression (61). The expressions of Dnmt3a in this region were also investigated in depression models. In one study, the Dnmt3a expressions in the hippocampus of depressed rats (maternal deprivation) were increased, which could be alleviated by antidepressant treatment with escitalopram (55). Specifically, this alteration was found in the dorsal hippocampus but not ventral hippocampus, accompanied by increased DNA methylation. Additionally, in another study, injection of DNA methylation inhibitors into the hippocampus of the depressed mice could decrease the levels of DNA methylation in the hippocampus, and it had antidepressant-like effects (confirmed *via* FST and TST) (62). Whether this antidepressant effect is related to the inhibition of Dnmt3a activity in the hippocampus needs further study.

Compared to DNMT3A, DNMT3B is little studied due to its early expression during brain development and may play a less important role than DNMT3A. However, one study suggests DNMT3B may also change in depression, paralleling DNMT3A. In this study, the Dnmt3b expressions in the prefrontal cortex of depressive mice were higher than those in normal controls. After treatment with the antidepressant imipramine, the Dnmt3b expression in prefrontal cortex was decreased compared with that in the group without treatment (9).

In summary, given the contradictory observations among the studies of DNMT3A and DNMT3B in depression patients and depression-like animal models, it is still early to draw a conclusion based on the available data. However, the above-mentioned data do support the correlation and some causal relationship between DNA methylation and depression signs in patients or endophenotypes in animal models. The discrepancy may be caused by different experimental methods, depression models, sex and age or environmental changes. Also, we believe that DNA methylation is a dynamic process, and there are individual differences on its interaction with genes.

### DNMT3L

DNMT3L has no direct catalytic effect, but it can be directly combined with DNMT3A and DNMT3B, and their enzyme activities can be increased by about 1.5–3 times (18). Some studies showed that DNMT3L increased the enzyme activity of DNMT3A more than DNMT3B (63). The only report on DNMT3L expression in depression patients showed the expression of DNMT3L in blood samples of patients with depression was not significantly different from that of psychiatrically healthy controls (51).

However, DNMT3L may play an important role during neurodevelopment. *DNMT3L* is located on chromosome 21, and its overexpression may cause the hypermethylation and alter the gene expression that controls the neurodevelopment (64). Ethanol exposure could decrease the Dnmt3l mRNA in the amygdala of adolescent rats (65). There are few data about the expression of DNMT3L in the developing brain. The RNA expressing data from the Allen Institute showed that the expression of DNMT3L in the human brain increased from gestational age of 16 weeks until adult. Though at low levels, it distributed broadly, including cerebral cortex, cerebellar cortex, hippocampus, striatum, thalamus, and amygdala (24). In primate brain, the Dnmt3l distributed mainly in the ventricular and subventricular zone, and limited to hippocampus and amygdala after birth (23). Opposite to DNMT3L, DNMT3A, and DNMT3B expressions peaked around gestational age of 16 weeks and decreased after until adult (24). In primate brain, they mainly distributed in the ventricular zone, subventricular zone, hippocampus and amygdala, where the neural stem cells locate (23). Consistent with these observations in human and primates, a recent study in rats showed that the mRNA expression of Dnmt3l in the hypothalamus and hippocampus followed a pattern opposite to the other Dnmts, that was low expression in newborns and increasing expression with age (66). This indicates DNMT3L may function differently compared with other DNMTs, important in neurogenesis and neural plasticity, which are essential processes during brain maturation, a stage when the prevalence of early-onset depression is high. A summary of the DNMTs changes in depression patients and animal models is listed in **Table 1**.

**Table 1:** DNMTs Expression in Depression Patients and Animal Models.

Sample	Tissue	Phenotype	DNMT	Reference
Suicide patients with MDD	Frontal cortex, Amygdala, Paraventricular nucleus	MDD	Frontal cortex: DNMT1 mRNA decreased; DNMT3A mRNA no change; DNMT3B mRNA increased. Amygdala: DNMT1 mRNA decreased; DNMT3A mRNA no change; DNMT3B mRNA decreased. Paraventricular nucleus: DNMT1 mRNA decreased; DNMT3A mRNA no change; DNMT3B mRNA increased	(6)
Patients with MDD and BPD	Peripheral white blood cells	MDD, BPD	DNMT1 mRNA decreased: DNMT3B mRNA increased; DNMT3A mRNA no change; DNMT3L mRNA no change	(51)
Patients with MDD	Dorsolateral prefrontal cortex, Cingulate cortex, Leucocyte	MDD	Dorsolateral prefrontal cortex: DNMT3B mRNA increased; DNMT3A mRNA no change; DNMT1 mRNA no change	(7)
Chronic defeat stress in mice	Nucleus accumbens	Depression-like behaviors	Dnmt3a mRNA increased	(59)
Rat depression model	Hippocampus	Depression-like behaviors	DNA methylation inhibitors induced antidepressant-like effects	(62)
Flinders Sensitive Line genetic rat model of depression	Prefrontal cortex	Reversing depressive-like behaviors	Increased Dnmt1 and Dnmt3a mRNA expression were decreased after SSRI treatment	(31)
Subchronic variable stress in mice	Nucleus accumbens	Depression-like behaviors	Dnmt3a mRNA increased	(60)
Dnmt1 and Dnmt3a knockout mice	Forebrain	Anxiolytic and antidepressant-like properties	Dnmt1 knockout	(52)
Adult mice with chronic social defeat stress	Medial Prefrontal Cortex	Anxiety-like behaviors	Dnmt3a mRNA decreased	(57)
Prenatally stressed dams in rats	Hippocampus	Reversing depression-like behaviors	Increased Dnmt1 mRNA and protein expression were decreased after Genipin treatment	(53)
Rat pups with maternal separation	Hippocampus	Reversing depression-like behaviors	Increased Dnmt1 and Dnmt3a mRNA expression were decreased after citalopram treatment	(67)
Stress model of depression in rats	Dorsal and ventral hippocampus, Prefrontal cortex	Depression-like behaviors	Dnmt3a and Dnmt3b protein expression increased in the prefrontal cortex, reversed by imipramine treatment	(9)
Two-hit stress model in mice	Hippocampus	Depression-like behaviors	Dnmt1 mRNA increased	(55)

## A Model of Gene-Environment Interaction Through DNA Methylation

Many studies have suggested that early life adversity (ELA) such as maternal separation and child maltreatment is a major risk factor of MDD (68). ELA as a stressor causes abnormal nervous system arousal during early neurodevelopment. It continually activates the amygdala, which then sends signals to the hypothalamus to activate the HPA axis for the release of glucocorticoids, which functions through GRs and mineralocorticoid receptors (MRs) to regulate the body’s response to stress (69). These ELA events may cause HPA hyperactivity and GR resistance, which are correlated with later life psychiatric disorders including MDD. ELA is also associated with structural changes in different brain areas, including amygdala, hippocampus, and prefrontal cortex (70, 71). The correlation of DNA methylation in GR gene *NR3C1* with ELA and depression have been reported in several studies in patients (43, 72–78), with most cases showing increased methylation in the promoter region of *NR3C1*.

**Figure 1:**
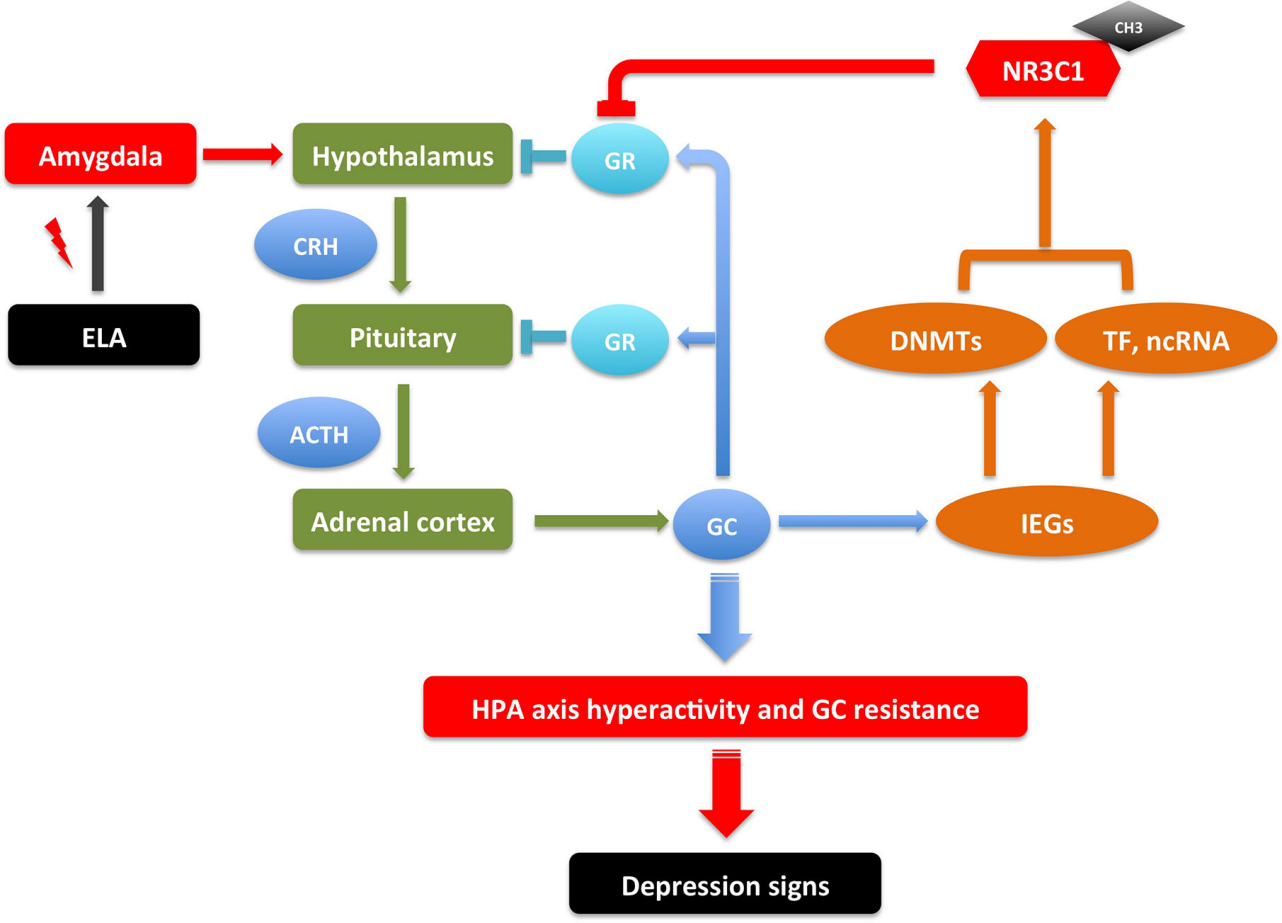
An illustrative model of gene-environment interactions underlying depressive behaviors through *NR3C1* methylation. ELA events send signals to neurons in amygdala, which then send signals to hypothalamus to activate the HPA axis to release GC. In a feedback loop, GC activates GR in hypothalamus and pituitary to inhibit the HPA axis activation; it also activates IEGs that increase the transcription of DNMTs, TFs, and ncRNAs, which together up-regulate the DNA methylation in the promoter of *NR3C1*, inhibiting the expression of GR. These serial signals transduction results in HPA axis hyperactivity and GR resistance, which may cause increased inflammation and imbalanced neurotransmitters, therefore the susceptibility of depression. The depression signs in patients include depressed mood, anhedonia, lack of motivation, psychomotor changes (agitation/retardation), psychotic changes (delusion, hallucination), and cognitive changes (verbal memory, working memory, executive function). The depression-like signs in animal models include anhedonia-like behavior, despair-like behavior, anxiety-like behavior, locomotor changes, and cognitive changes (97–100). This is a reversible process. The depression signs can be treated by regaining the GR activity with drugs that interfere with DNA methylation or GR expression (69, 91–96). ACTH, Adrenocorticotropic hormone; CH3, DNA methylation; CRH, Corticotropin-releasing hormone; DNMTs, DNA methyltransferases; ELA, early life adversity; GC, glucocorticoids; GR, glucocorticoid receptor; HPA, hypothalamic–pituitary–adrenal; IEGs, immediate early genes; ncRNA, non-coding RNA; TF, transcription factor.

Consistent with the observations in patients, multiple studies have found the increased DNA methylation in *Nr3c1* and decreased GR expression in hippocampus of ELA mice and rats (79–85). Correspondingly, the DNA methylation in *Crh* promoter was decreased and its expression increased in the hypothalamus of ELA models. The other genes in the HPA axis such as AVP and ACTH were also reported to have similar DNA methylation changes in ELA models (44, 86–88). Supporting this machinery, the DNMTs were also found to be increased in these ELA models (89, 90). However, the global methylation changes affect many other genes resulting both hypermethylation and hypomethylation. Taken together, the DNA methylation changes of genes in HPA axis connect the ELA with neural activity, which may increase the susceptibility of the later life psychiatric disorders though HPA axis hyperactivity and GC resistance, associated with increased inflammation and imbalanced neurotransmitters (69, 91–96). It thus makes a model of gene-environment interaction for the pathogenesis of MDD (**Figure 1**), where the DNA methylation changes of genes in HPA axis are adaptive to the stress stimulation, which are reversible once the stress is removed; it induces depression though if the stressor stays for a long period. However, this could be oversimplified since there are individual differences on susceptibility and resilience reported due to different ELA types, sex, age, developmental time, and genetic background (69). Also, the DNA methylation mechanism in the regulation of gene expression is complicated. How does ELA change the expression of DNMTs? How does DNMTs specifically regulate genes in HPA axis? And how does HPA axis changes lead to increasing incidence of MDD? All these questions need thorough investigations.

## Discussion

The depression studies have a long history, but the study of DNA methylation and DNA methyltransferase is still in its infancy. At present, many studies have shown that changing the DNA methylation state in brain can alleviate the depression-like behaviors in animal experiments. For example, Sales et al. found that systematic injection of DNA methylation inhibitors (5-AzaD and RG108) into depressed mice could rescue the despair-like behavior and effectively relieve hypermethylation in hippocampus and prefrontal cortex caused by stress (101). Xing et al. also found that inhibition of DNMT activity in prefrontal cortex could play an antidepressant role (102). It may be related to the changes of DNMTs expressions and the increases of DNA methylation in these regions. The methylation levels of BNDF coding gene in hippocampus and prefrontal cortex were increased in patients with depression, and the results of autopsies showed atrophy in hippocampus and prefrontal cortex (103, 104). These changes are consistent with the changes of DNMTs in the corresponding brain regions, but the causal relationship between them is still not clear.

At present, we can confirm that DNMTs are involved in the pathogenesis of depression, but we do not know what causes the DNMTs changes, and how the change of DNMT expression in brain tissue affects the expression of specific genes. Pacaud et al. believe that transcription factors bind to DNMTs, thus exerting biological effects (105). Combining the changes of gene expression and DNMTs mentioned in this review, we can speculate as follows: 1) there are some tissue specific or cell specific transcription factors that can bind to the promoter region of related genes such as *P11*, *BDNF*, and *SLC6A4*, and DNMTs can bind these transcription factors and regulate the expression of the targeting genes; 2) DNMTs may function through catalyzing DNA methylation to regulate the gene expression, assisted by the transcription factors, miRNA or lncRNA to get to the specific targeting genes; 3) environmental changes may activate some IEGs, which in turn activate or inhibit the DNMTs expression; 4) the current depression model is based on the loop formed between several brain regions of the limbic system, and the completion of the loop function depends on the normal secretion, release and transport of excitatory and inhibitory neurotransmitters. Therefore, whether the changes of DNMTs in these related brain regions affect the expression of these neurotransmitters is also a question that needs to be further investigated.

Other studies have shown that gender differences exist for the expression of MeCP2, SLC6A4, and other genes in blood samples of depression patients (106), but it is not clear whether it is related to the sex difference in the expression of DNMTs mentioned in this review. Kolodkin et al. found that Dnmt3a expression in the amygdala of rats at birth had a sex difference, and the expression of Dnmt3a in the female rats was higher than that in the male rats, but the gender difference vanished at the 10th day of birth (107). The prefrontal cortex, nucleus accumbens, amygdala, and hippocampus form reward loops closely related to addiction and mood regulation. According to the sex difference of Dnmt3a expression in nucleus accumbens and amygdala and the characteristics of decreased estrogen and progesterone levels in women during menopausal period (108), we can infer: 1) estrogen can affect the expression of DNMT3A in amygdala; 2) the high incidence of depression in women may be due to the inhibitory effect of estrogen on DNMT3A expression in amygdala, which is higher than that of androgen; 3) the sex difference in DNMT3A expression in the nucleus accumbens and DNMT1 in the amygdala may be due to the different depression patterns in men and women; 4) the higher incidence of menopausal depression in women may be due to the decrease of estrogen, so the inhibitory effect on DNMT3A is reduced.

To test the above conjectures, we need following studies: 1) to explore the mechanism of DNMT3L regulating the activities of DNMT3A and DNMT3B under different conditions; 2) to observe the expression of DNMT1, DNMT3A, DNMT3B, and DNMT3L in different brain regions and the effect of estrogen on them under physiological conditions; 3) under physiological conditions, to observe the process of DNA methylation dynamically and capture the changes of DNMT expression in different brain regions at different time points, then record the time sequence of DNMT expression changes; 4) to study the expression and activity of DNMT at cellular level; 5) to study the methylation process of DNMTs to specific gene promoter sequences at molecular level; 6) to start with the genes with altered expression in depression, use DNMTs and DNA methylation as the intermediary to find the signal pathway that regulates the expression of specific genes.

It is difficult to obtain the brain tissue of patients with depression, which brings some difficulties to the exploration of the pathogenesis of depression. However, with the invention of methods to noninvasively visualize epigenetic processes *in vivo*, such as PET radiotracers for histone deacetylases (HDACs) (109, 110), we can expect the development of PET radiotracers for DNMTs too and dynamic DNA methylation changes could then be traced in depression patients. Recent technical advancement on induced pluripotent stem cells may help to solve this problem by making cellular model by using the fibroblasts from depression patients. Besides this, by making use of other molecular techniques such as CRISPR, methylation editing, optogenetics, and single cell technologies, we could work on these questions and will better understand the mechanisms of DNMTs in the pathogenesis of depression.

## Author Contributions

JL: conceived the topic of manuscript. JL and ZD: collected the data and carried out the main analysis. All authors contributed to the article and approved the submitted version.

## Funding

This work was supported by grants to JL from National Natural Science Foundation of China (81771229).

## Conflict of Interest

The authors declare that the research was conducted in the absence of any commercial or financial relationships that could be construed as a potential conflict of interest.
